# Utilization of mechanocatalytic oligosaccharides by ethanologenic *Escherichia coli* as a model microbial cell factory

**DOI:** 10.1186/s13568-020-0965-4

**Published:** 2020-02-03

**Authors:** Tao Jin, Mats Käldström, Adriana Benavides, Marcelo D. Kaufman Rechulski, Laura R. Jarboe

**Affiliations:** 1 Department of Chemical and Biological Engineering, Iowa State University, 4134 Biorenewables Research Laboratory, 617 Bissell Rd, Ames, IA USA; 2 Department of Heterogeneous Catalysis, Max-Planck-Institut für Kohlenforschung, Mulheim an der Ruhr, Germany; 3 BioMAP REU Program, Ames, IA USA

**Keywords:** Biofuels, Fermentation, Coli, Mechanocatalytic, Oligosaccharides

## Abstract

Mechanocatalysis is a promising method for depolymerization of lignocellulosic biomass. Microbial utilization of the resulting oligosaccharides is one potential route of adding value to the depolymerized biomass. However, it is unclear how readily these oligosaccharides are utilized by standard cell factories. Here, we investigate utilization of cellulose subjected to mechanocatalytic depolymerization, using ethanologenic *Escherichia coli* as a model fermentation organism. The mechanocatalytic oligosaccharides supported ethanol titers similar to those observed when glucose was provided at comparable concentrations. Tracking of the various oligomers, using maltose (alpha-1,4) and cellobiose (beta-1,4) oligomers as representative standards of the orientation, but not linkage, of the glycosidic bond, suggests that the malto-like-oligomers are more readily utilized than cello-like-oligomers, consistent with poor growth with cellotetraose or cellopentaose as sole carbon source. Thus, mechanocatalytic oligosaccharides are a promising substrate for cell factories, and microbial utilization of these sugars could possibly be improved by addressing utilization of cello-like oligomers.

## Introduction

There is extensive interest in the production of fuels and chemicals from biomass, as opposed to petroleum. The use of biomass-derived carbon, as opposed to petroleum-derived carbon is attractive for a variety of reasons, including but not limited to sustainability and energy security. Microbial utilization of biomass has historically focused on the cellulose-derived hexose sugar components of sugar crops, such as corn, beets and sugar cane (Jin et al. 2017). However, focus has expanded to include the utilization of the hemicellulose and lignin components of lignocellulosic biomass, such as corn stover, switchgrass, and woody biomass (Guerriero et al. 2016; Tye et al. 2016).

**Fig. 1:**
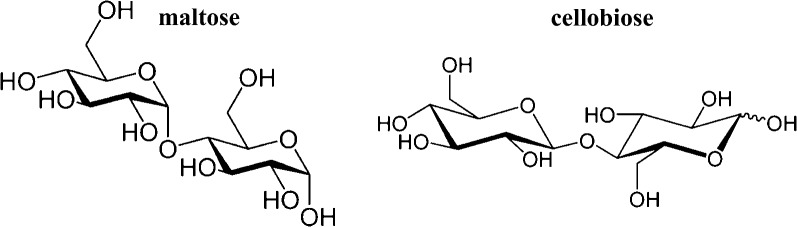
Structure of two representative sugar dimers, maltose and cellobiose. Malto-sugar oligomers have an alpha orientation of the 1–4 bond and cello-sugar oligomers have a beta orientation of the 1–4 bond

Utilization of lignocellulosic biomass, either as a feedstock for a catalytic process or as a carbon and energy source for microbial cell factories, requires depolymerization of the biomass. A variety of methods have been developed for this depolymerization, including but not limited to: enzymes (Dutta and Wu 2014), hydrolysis (Loow et al. 2016), ionic liquids (Badgujar and Bhanage 2015), thermochemical processing (Shen et al. 2015), and organic solvents (Zhang et al. 2016). Another promising method of biomass depolymerization is mechanocatalytic processing (Hick et al. 2010; Jerome et al. 2016; Kaldstrom et al. 2014a, b; Rechulski et al. 2015; Schneider et al. 2016; Meine et al. 2012). In this approach, acid-impregnated cellulose or biomass is subjected to a relatively short, on the scale of minutes to hours, period of ball milling, resulting in the conversion to water-soluble oligomers of xylose, glucose and the anhydrosugar levoglucosan (Meine et al. 2012). Structures of two representative sugar oligomers, maltose and cellobiose, are shown in Fig. 1. It has been proposed that the nature of the mechanocatalysis process results in non-stereospecific bonds between these sugar units (Meine et al. 2012), including possible 1,6 linkages (Shrotri et al. 2013). The use of the sugar oligomers produced by the mechanocatalytic biomass depolymerization as the carbon and energy source for fermentation has not, to the best of our knowledge, been previously reported.

The goal of this paper is to assess the ability of microbial biocatalysts to use the mechanocatalysis product, in the absence of an explicit oligosaccharide hydrolysis step, as a substrate for the production of biorenewable fuels and chemicals, using ethanologenic *Escherichia coli* KO11 + *lgk* as a model fermentation organism. This organism and strain was selected due to its previously demonstrated ability to utilize pentose sugars, hexose sugars and a wide range of depolymerized biomass types (Jarboe et al. 2007) and because it has been engineered to utilize the anhydrosugar levoglucosan (Layton et al. 2011), an abundant component of the mechanocatalytically depolymerized sugars (Meine et al. 2012).

## Materials and methods

### Strains and growth media

Construction of *E. coli* strain KO11 + *lgk* was previously described (Layton et al. 2011). Strains were grown in Luria Broth supplemented with glucose, mechanocatalytic sugars or in MOPS minimal media (Neidhardt et al. 1974) with monomeric sugars as indicated. Media was adjusted to pH 7.0 prior to inoculation. Cells were grown at 37 °C in 20 mL of media containing 40 μg/mL of chloramphenicol in 50 mL tubes with shaking at 200 rpm. Malto- and cello-oligomers were purchased from Sigma-Aldrich.

### Production of sugars

Sugars were produced by ball milling of α-cellulose using sulfuric acid as catalyst, as previously described (Meine et al. 2012).

### Measurement of sugars and ethanol

One ml of fermentation broth was taken every 24 h and centrifuged at 13,000 rpm. The supernatant was used for measurement of sugar consumption and ethanol production. Glucose, xylose and levoglucosan were measured by Ion Exchange Chromatography ICS 5000 system (Sunnyvale, CA) equipped with a CarboPac™ PA 20 analytical column and a CarboPac™ PA 20 guard column. The flow rate of 0.01 M NaOH was 0.5 mL/min. The pulsed amperometric detector was used to characterize sugar compositions. Ethanol production was measured by a Bruker 450 GC-FID (Varian Inc., Walnut Creek, CA) equipped with a Zebron ZB-WAXplus capillary column (Phenomenex, Torrance, CA). The samples were injected at a split ratio of 20:1 with the injection temperature at 200 °C. The flow rate of helium was 25 mL/min. The oven temperature was 35 °C for 5 min and then increased to 130 °C at the rate of 10 °C/min.

Malto- and cello-oligomers were measured by HPLC using the Prevail™ carbohydrate ES S4 column at 30 °C. The mobile phase was 65 vol% acetonitrile and 35 vol% water, with a flowrate of 0.8 mL/min. An evaporative light scattering detector (ELSD) was used at 35 °C with a nitrogen flow of 1.2 mL/min and a detector gain of 4. When necessary, samples were diluted with distilled water to fit the calibration curve. Peak identification and quantification were conducted using external standards.

## Results

### Oligosaccharides produced by mechanocatalytic biomass depolymerization support similar ethanol titers as those seen with pure glucose

**Fig. 2:**
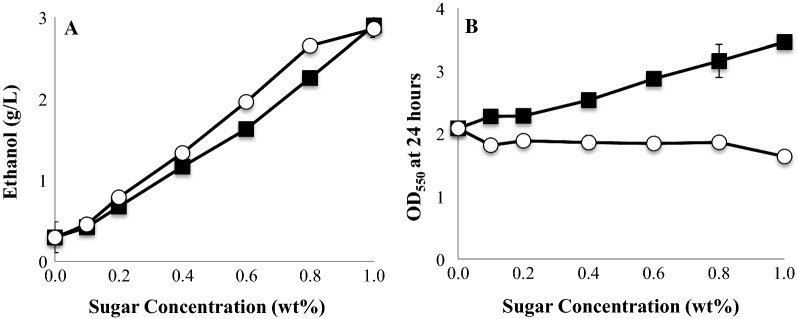
Mechanocatalytic sugars (filled squares) support ethanol production with titers similar to those observed for pure glucose (white circles). Fermentation was performed with ethanologenic *E. coli* KO11 + *lgk* in LB for 24 h at 37 °C, 200 rpm. **a** Ethanol production; **b** growth. Data is the average of three biological replicates, with error bars indicating one standard deviation

Mechanocatalytic sugars produced from α-cellulose with sulfuric acid as catalyst were used as the fermentation substrate for ethanologenic *E. coli* KO11, with pure glucose provided as a control (Fig. 2a). Ethanol titers were similar between the two sugar types at all tested concentrations (P > 0.05). It was observed that 3.0 g/L ethanol was produced within 24 h from 1.0 wt% of each type of sugar.

While ethanol titers were indistinguishable between the two types of sugars, microbial growth differed substantially (Fig. 2b). Specifically, cultures containing the mechanocatalytic oligosaccharides showed a dose-dependent increase in biomass production across the entire tested range, while no such effect was observed for glucose. This difference may be due to the occurrence of overflow metabolism during glucose utilization, but not during mechanocatalytic oligosaccharide utilization (Xu et al. 1999; Basan et al. 2015). In such a scenario, partially metabolized glucose would be discarded as acetate instead of complete processing through ATP-yielding respiratory reactions, with the accumulation of acetate and the lower ATP yield both negatively impacting biomass production relative to the non-glucose condition. This proposed explanation is consistent with reports that decreasing the glucose uptake rate can decrease occurrence of overflow metabolism (Jung et al. 2019).

### Malto-sugar oligomers are more readily utilized than cello-sugar oligomers

Having demonstrated that the complex mixture of mechanocatalytic sugars can be utilized by *E. coli* as effectively as glucose, we next aimed to determine which sugars were being readily utilized and which sugars require metabolic engineering, such as the introduction of foreign pathways or expression tuning, for sufficient utilization.

Here, maltose and cellobiose oligomers were used as standards for assessment of the various oligosaccharides. These two glucose oligomers differ in their orientation of their 1,4-glycosidic bonds. Malto-oligomers are in the α orientation, while cello-oligomers are the β orientation (Fig. 1). As mentioned above, the mechanocatalysis process is expected to result in non-stereospecific bonds between the sugar monomers (Meine et al. 2012). The existence of other linkages within this pool of oligosaccharides, such as 1,6, has been reported (Shrotri et al. 2013). The malto- and cello-oligomers used here are expected to represent the orientation of the glycosidic bond but not the linkage. The rich medium in which these experiments were performed contains yeast extract and peptone, and thus also contained detectable amounts of these sugar oligomers (Table 1).

**Table 1:** Concentration (mg/L) of the major media components during fermentation of 1.0 wt% mechanocatalytic oligosaccharides in LB media by ethanologenic *E. coli* KO11 + *lgk* at 37 °C, 200 rpm

Sugars	LB media	+ 1.0 wt% mechanocatalytic sugars			
		Initial	24 h	48 h	72 h
Monomers					
Glucose	n/a	270	n.d.	n.d.	n.d.
Xylose	n/a	550	n.d.	n.d.	n.d.
Levoglucosan	n/a	16	33	10	7
Malto-oligomers					
-ose	n.d.	n.d.	n.d.	n.d.	n.d.
-triose	110	410	490	370	98
-tetraose	120	190	240	170	110
-pentaose	130	300	220	130	130
-hexaose	110	690	570	560	570
-heptaose	140	110	130	160	140
Total	610	1700	1650	1390	1048
Cello-oligomers					
-biose	n.d.	n.d.	n.d.	n.d.	n.d.
-triose	98	170	630	500	700
-tetraose	160	320	460	400	500
-pentaose	160	120	130	120	130
-hexaose	170	230	170	170	160
-heptaose	180	120	160	170	160
Total	768	960	1550	1360	1650
Total, all sugars	1400	3500	3200	2800	2700
Products					
Dry cell mass	n/a	0.016	1.15 ± 0.03	1.35 ± 0.03	1.38 ± 0.03
Ethanol	n/a	0	2900 ± 200	2600 ± 100	2150 ± 40

Malto- and cello-oligomers are expected to represent the orientation of the glycosidic bond, but not necessarily the linkage. Glucose, xylose and levoglucosan were measured by IEC, other sugars by HPLC. Ethanol was measured by GC and dry biomass content was estimated from OD_550_ values

*n/a* not determined, *n.d.* none detected

As expected, the monomeric glucose and xylose were completely consumed within the first 24 h (Table 1). Low concentrations of levoglucosan persisted throughout the 72-h fermentation.

Depolymerization of malto-oligomers by *E. coli* is well-established (Boos and Shuman 1998) and is supported by our measurements (Table 1). Specifically, the abundance of maltotriose- and maltotetraose-linked oligomers increased during the first 24 h, consistent with production of these oligomers by enzymatic depolymerization of higher-order oligomers at a rate that exceeds their rate of consumption. Depolymerization and consumption of the malto-like-oligomers is further supported by the continual decrease of the malto-oligomer pool over the course of the fermentation, with 60% of the original 1700 mg/L being consumed within 72 h (Table 1).

The data suggests that a similar depolymerization cascade is occurring with the cello-like oligomers, but at a lower rate. Specifically, the concentration of cellotriose- and cellotetraose-like oligomers increased at a rate of 6 ± 3 and 2 ± 1 mg/L/h, respectively, over the course of the experiment. This increase in concentration suggests continual release of these sugars from higher-order oligomers at a rate exceeding their subsequent microbial consumption. But unlike the malto-like-oligomers, the total pool of cello-like-oligomers increased over the course of the experiment.

The total concentration of sugar oligomers expected to be provided by 1.0 wt% of mechanocatalysis product is 10 g/L, but the detection methods used here only account for 3.5 g/L. Considering that the rich media contributes 1.4 g/L of sugars, roughly 80% of the mass of the mechanocatalytic oligosaccharides is not accounted for in Table 1. However, it is clear that the malto-like-oligomers are being depolymerized and consumed more readily than the cello-like-oligomers.

Over the first 24 h, 2.9 g/L of ethanol were produced. Given that cultures with no added sugar produced 0.3 g/L of ethanol over a similar time period (Fig. 2a), nearly 90% of the ethanol production can be attributed to microbial utilization of the mechanocatalysis product. This ethanol product was associated with the consumption of only 300 mg/L of measured sugars (Table 1), further emphasizing the fact that the measurement techniques used here are only capturing a minority of the total sugars. The decrease in ethanol titer after the 24-h sample is likely due to evaporation of the ethanol from the fermentation media and is consistent with other reports (Ohta et al. 1991).

**Fig. 3:**
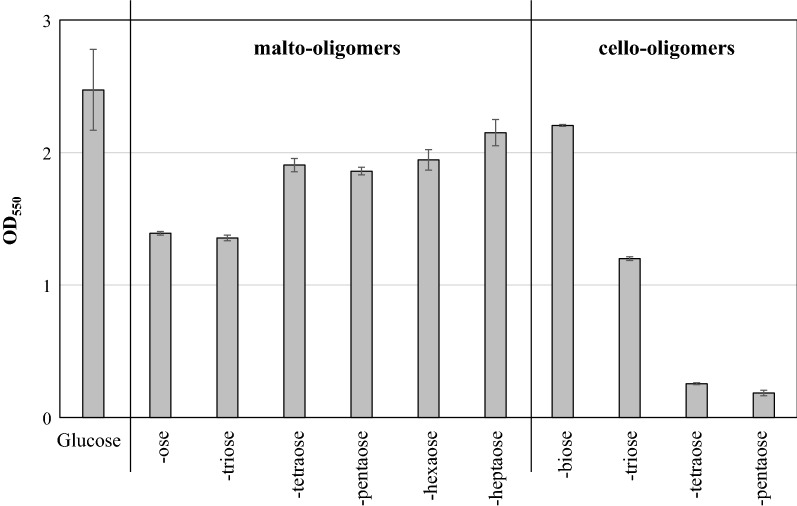
Growth on pure malto- and cello-oligomers as sole carbon source. *E. coli* KO11 + *lgk* was grown in MOPS minimal media containing the indicated carbon source at 37 °C with shaking at 200 rpm for 48 h. Glucose, all of the malto-oligomers and cellobiose were added to 0.50 wt%. Cellotriose, cellotetraose, and cellopentaose were added at 0.10 wt%. Values are the average of three biological replicates, with error bars indicating one standard deviation

The results above indicate that cello-like-oligomer consumption by *E. coli* is relatively poor compared to malto-like-oligomer consumption. While the utilization of malto-oligomers and cellobiose have been well-characterized in *E. coli* (Boos and Shuman 1998; Hall and Faunce 1987), the utilization of higher-order cello-oligomers is relatively undercharacterized (Andersen et al. 1999). Consistent with the measurement of oligomer abundance during utilization of the mechanocatalysis product, studies performed in defined minimal media with pure oligomers as substrate support the conclusion that malto-oligomers are more readily utilized than cello-oligomers (Fig. 3). Specifically, each of the tested malto-oligomers, cellobiose and cellotriose all supported growth to an OD_550_ of at least 1.0. Contrastingly, cellotetraose and cellopentaose did not, with final OD_550_ values less than 0.3.

## Discussion

The theoretical yield of ethanol from glucose using strain KO11 is 2 mol ethanol per mole of glucose (Jarboe et al. 2007) and thus the expected ethanol titer during utilization of 1.0 wt% glucose is 5.1 g/L. Observation here of only 3.0 g/L is likely due to the absence of pH control and the small culture volume due to the small amounts of mechanocatalytic sugars available. The fact that the mechanocatalytic sugars support production of ethanol at similar titers to pure glucose is promising for their use as a substrate for the production of a wide range of fuels and chemicals. Compared to the rapid consumption of glucose and xylose, slow decline of the concentrations of levoglucosan can probably be attributed to a combination of two factors: (1) the relatively high K_m_ of levoglucosan kinase (LGK) for levoglucosan of approximately 1.4 wt% (Bacik et al. 2015) and (2) the production of levoglucosan from the depolymerization of higher-order oligomers. This depolymerization could be similar to the previously described acid-mediated hydrolysis of cellobiosan to cellobiose and levoglucosan (Helle et al. 2007), though the temperature and pH used here are much milder than those used for characterizing said hydrolysis.

Sustained growth of the total pool of cello-like-oligomers was observed throughout the experiment. This most likely can be attributed to the lack of sensitivity of our experimental methods to measurement of some of the higher-order cello-oligomers, and thus they were not measured as a part of the cello-oligomer pool until they were sufficiently depolymerized.

These results demonstrate that oligosaccharides produced by mechanocatalytic depolymerization of cellulose can be used as carbon source for fermentative bio-production, with product titers similar to those observed with pure glucose. The malto-like-oligomers were readily utilized by ethanologenic *E. coli* (Table 1), consistent with existing knowledge of *E. coli* metabolism (Boos and Shuman 1998). The cello-like-oligomers were also utilized, but at a lower rate. The decreased utilization of the cello-like-oligomers was also apparent during utilization of pure oligomers (Fig. 3).

It is important to increase the efficiency of the cellulose deconstruction in order to increase the substrate accessibility for microbial utilization. For example, the synergetic action of endo-1,4-β-glucanases, exo-1,4-β-glucanases and β-glucosidases can convert cellulose to glucose (Horn et al. 2012). Lytic polysaccharide monooxygenases (LPMO) have been proved as effective enzymes in degradation of cellulose as well (Hemsworth et al. 2015; Johansen 2016). In additional to enzymatic hydrolysis of cellulose, pretreatment under such as alkaline conditions (Knill and Kennedy 2003) can also achieve this goal. Moreover, metabolic engineering efforts to improve utilization of cello-like oligomers could be also a promising strategy for improving microbial utilization of these oligosaccharides.

## Data Availability

The data on which the conclusions are made are all presented in this paper.
